# Effects of continuous versus intermittent enteral feeding on feeding tolerance in critically ill adults: a systematic review and meta-analysis

**DOI:** 10.3389/fmed.2026.1738036

**Published:** 2026-01-21

**Authors:** Yahui Wang, Meng Li, Wanqian Zhao, Xiaofeng He, Shaoling Xiao

**Affiliations:** 1 School of Nursing, Changzhi Medical College, Changzhi, China; 2 Pharmacy Intravenous Admixture Services, Heping Hospital Affiliated to Changzhi Medical College, Changzhi, China; 3 Institute of Evidence-Based Medicine, Heping Hospital Affiliated to Changzhi Medical College, Changzhi, China; 4 Department of Science and Education, Heping Hospital Affiliated to Changzhi Medical College, Changzhi, China

**Keywords:** enteral nutrition, continuous enteral feeding, intermittent enteral feeding, critical illness, meta-analysis

## Abstract

**Objectives:**

This study aims to comprehensively evaluate the impact of continuous versus intermittent enteral feeding regimens on feeding tolerance in critically ill patients during clinical practice.

**Method:**

A systematic search was conducted in the databases of PubMed, Web of Science, Embase, and the Cochrane Library for studies published up to June 30, 2025, to identify clinical studies evaluating the effects of continuous versus intermittent enteral feeding strategies in critically ill patients. The primary outcomes were defined as the incidence of gastrointestinal intolerance events, including diarrhea, constipation, vomiting, gastric residual volume, and abdominal distension, as well as feeding-related complications such as aspiration and pneumonia. Secondary outcomes included ICU mortality, length of ICU stay, and achieved energy intake.

**Results:**

A total of 3,517 studies were initially identified, with 17 randomized controlled trials meeting the eligibility criteria and included in the meta-analysis. The results demonstrated that continuous enteral feeding was associated with an elevated risk of constipation (RR = 1.40, 95% CI = 1.01–1.95). No statistically significant differences were observed between the two feeding regimens for the outcomes of diarrhea, vomiting, gastric residual volume, abdominal distension, aspiration, ICU mortality, and length of ICU stay. Subgroup analyses based on intervention duration (<7 days versus ≥7 days) indicated a higher risk of constipation with continuous feeding in the subgroup with an intervention duration <7 days (RR = 2.55, 95% CI = 1.15–5.69), whereas no significant difference was found in the subgroup with an intervention duration ≥7 days (RR = 1.25, 95% CI = 0.89–1.75). The included studies carried some risk of bias. According to the GRADE approach, the overall certainty of the evidence for all outcome measures was low or very low.

**Conclusion:**

In critically ill patients, continuous enteral feeding is associated with an elevated risk of constipation, and this risk is particularly elevated during short-term interventions (<7 days). No significant differences were observed between the two feeding regimens for other clinical outcomes. Given the generally low quality of evidence and the small sample sizes of the included studies, these conclusions should be interpreted with caution. Future large-scale, high-quality studies with long-term follow-up are necessary to further validate the efficacy of intermittent enteral feeding in alleviating gastrointestinal intolerance.

**Systematic review registration:**

https://www.crd.york.ac.uk/PROSPERO/view/CRD420251145362, identifier CRD420251145362.

## 1 Background

Malnutrition is relatively common among critically ill patients, with a reported incidence rate ranging from 39 to 50% (1, 2). Adequate nutritional intake is a critical determinant of survival. In critically ill patients, malnutrition exacerbates disease severity and is associated with increased mortality. Nutritional support is often initiated within the first 48 h of intensive care unit (ICU) admission for patients identified with nutritional risk (3). Enteral nutrition maintains intestinal barrier function, modulates immune responses, and reduces disease severity (4). These physiological benefits make it the preferred nutritional support strategy. Notably, enteral nutrition demonstrates beneficial effects in reducing infectious complications and facilitating recovery, yet it may concurrently elevate the risk of gastrointestinal intolerance (5, 6). Gastrointestinal intolerance encompasses elevated gastric residual volume (≥250 ± 50 mL), vomiting, absent bowel sounds, abdominal distension, or diarrhea (7). Gastrointestinal intolerance may lead to interruption of treatment, and in severe cases, it can increase the risk of infection, prolong the duration of mechanical ventilation, and raise mortality risk (8, 9). Therefore, improving enteral nutrition tolerance is critical for enhancing survival rates among intensive care unit patients (10). Identifying an optimized enteral nutrition regimen to effectively alleviate gastrointestinal intolerance and improve clinical outcomes is an urgent and critical priority in critical care medicine.

Enteral nutrition can be administered through two primary methods: continuous feeding and intermittent feeding. Continuous feeding is defined as the uninterrupted administration of enteral nutrition via an electronic feeding pump at a constant rate over a specified duration (11), typically ranging from 16 to 24 h (12). Continuous enteral nutrition, delivered through prolonged, steady, and slow infusion of nutritional formula, can significantly improve the achievement of nutritional targets in mechanically ventilated patients (13). This method also helps reduce the risk of gastrointestinal complications, such as aspiration, and contributes to better glycemic control (14). However, from a physiological perspective, continuous enteral feeding does not align with the body’s natural eating rhythm. In contrast, intermittent feeding more closely resembles physiological rhythms. It refers to the administration of enteral nutrition 4–6 times per day, with each session delivered over 20–60 min via an infusion pump or gravity drip (11). This feeding pattern may promote gastric emptying and enhance digestive and absorptive functions through the modulation of gastrointestinal hormone secretion. Furthermore, intermittent feeding offers greater flexibility in clinical practice, facilitating patient participation in rehabilitation exercises (15). However, existing research has yielded inconsistent findings regarding the clinical efficacy of the two feeding regimens. McNelly et al. (16) reported that intermittent enteral feeding was associated with earlier achievement of target caloric intake, whereas Lee et al. (17) demonstrated more rapid attainment of nutritional goals with continuous feeding. Regarding gastrointestinal tolerance, Kadamani et al. (18) identified a higher occurrence of constipation with continuous feeding, while Banaei et al. (19) observed no significant increase in gastric residual volume and proposed that continuous feeding may be more suitable for critically ill patients.

In recent years, several meta-analyses have assessed the effects of different enteral feeding regimens in critically ill patients. However, these studies are subject to several methodological limitations. For example, the meta-analysis by Ma et al. (20) included a high proportion of Chinese-language studies, which may limit the generalizability of its conclusions. Heffernan et al. (21) incorporated a case–control study, thereby combining heterogeneous study designs and potentially compromising the validity of their pooled estimates. Thong et al. (22) conducted their literature search only up to April 2020, thus omitting relevant high-quality studies published subsequently. Qu et al. (23) did not utilize the most recent methodological quality assessment tools. Moreover, the review by Wu et al. (24) was constrained by a search across only three databases and further limited by small sample sizes.

Given the inconsistent conclusions and methodological limitations of existing studies, this systematic review and meta-analysis aims to conduct a comprehensive and methodologically rigorous synthesis of the evidence. By synthesizing the latest evidence, it will systematically compare the effects of continuous versus intermittent enteral feeding on gastrointestinal tolerance and related clinical outcomes in critically ill patients, thereby providing guidance for enteral nutrition clinical practice in this population.

## 2 Method

This meta-analysis adhered to the Preferred Reporting Items for Systematic Reviews and Meta-Analyses (PRISMA) guidelines (25), and the protocol was registered in the PROSPERO database (CRD42025114536).

### 2.1 Database and literature search strategy

A systematic search was conducted in the databases of PubMed, Web of Science, Embase, and the Cochrane Library for studies published up to June 30, 2025, to identify clinical studies evaluating the effects of continuous versus intermittent enteral feeding strategies in critically ill patients. The search strategy combined Medical Subject Headings (MeSH)/Emtree terms with free-text terms, including keywords such as “enteral nutrition,” “enteral feed*,” “nutrition* support,” “continuous,” “intermittent,” “bolus,” “intensive care units,” “critical care,” “adult,” “randomized controlled trial,” and related synonyms. For full details of the search strategy, please refer to Supplementary Table 1.

### 2.2 Inclusion and exclusion criteria

Studies were included if they fulfilled the following criteria: (1) Population: critically ill adults (≥ 18 years) receiving enteral nutrition in any ICU setting, with an intervention duration of at least 3 days (this threshold distinguishes transient physiological adaptation from clinically significant feeding intolerance); (2) Intervention: continuous enteral feeding regimen; (3) Comparison: intermittent enteral feeding regimen (bolus or sequential); (4) Outcome measures: reporting of at least one of the following outcomes: diarrhea, constipation, vomiting, gastric residual volume, abdominal distension, aspiration, pneumonia, ICU mortality, length of ICU stay, or achieved energy intake; (5) Study design: individually randomized, parallel-group randomized controlled trials (RCTs). Studies were excluded if they met any of the following criteria: (1) were published in languages other than English; (2) provided unclear descriptions of the feeding methods or were not designed to compare continuous versus intermittent enteral nutrition; (3) failed to report any of the prespecified primary or secondary outcomes; (4) were study protocols, review articles, cohort studies, case–control studies, conference abstracts, or book chapters.

### 2.3 Study selection

All database search results were imported into EndNote 21 (Clarivate Analytics, USA) for duplicate removal and study management. Two researchers (WYH and LM) independently screened the titles and abstracts to assess relevance. The full texts of potentially eligible studies were subsequently evaluated against the predetermined eligibility criteria. Any discrepancies were resolved through discussion with a third researcher (ZWQ) to reach a consensus.

### 2.4 Data extraction

For each included study, data extraction was performed according to the following predefined criteria: (1) general study information (author, publication year, country); (2) demographic and baseline clinical data (patient population, sample size, age, sex); (3) intervention protocols (caloric goal, feeding regimen, study period); and (4) outcomes (key findings). The primary outcomes were defined as the incidence of gastrointestinal intolerance events, including diarrhea, constipation, vomiting, gastric residual volume, and abdominal distension, as well as feeding-related complications such as aspiration and pneumonia. Secondary outcomes included ICU mortality, length of ICU stay, and achieved energy intake. In addition to quantitative results, we extracted the specific definitions of these outcomes as reported by the original investigators. Two independent researchers (WYH and LM) conducted the data extraction process. Any discrepancies were resolved through discussion with a third researcher (ZWQ) until consensus was reached. For studies reporting results as median and interquartile range (IQR) rather than mean and standard deviation (SD), we applied conversion formulas to estimate the mean and SD for data synthesis and statistical analysis (26, 27). GetData Digitizer version 2.20 software was used to extract the required data from graphs when numerical values were not provided in the text.

### 2.5 Quality assessment and certainty of evidence

Two researchers (WYH and LM) independently evaluated methodological quality. For RCTs, the Cochrane Risk of Bias 2 (RoB 2) tool was applied to assess five key domains: randomization process, deviations from the intended interventions, missing outcome data, measurement of the outcome, and selection of the reported result (28). The risk level was judged as high risk, some concerns, or low risk based on responses to the signaling questions within each domain.

The certainty of the evidence for critical outcomes was assessed using the Grading of Recommendations, Assessment, Development, and Evaluations (GRADE) framework, which involves a systematic assessment across five key domains: risk of bias, inconsistency, indirectness, imprecision, and publication bias (29). Through the GRADEpro GDT software, the evidence for each outcome was categorized into one of four predefined certainty levels: high, moderate, low, or very low.

### 2.6 Statistical analysis

All statistical analyses were performed using Stata 18.0. Dichotomous outcomes were relative risks (RR) and 95% confidence intervals (CI), and continuous outcomes as the standardized mean difference (SMD) and 95% CI. All studies were pooled using the inverse variance method, with a continuity correction of 0.5 applied to those containing zero events. Heterogeneity among studies was assessed using Cochran’s *Q* test and quantified by the *I*^2^ statistic, with values of 25, 50, and 75% indicating low, moderate, and high heterogeneity, respectively. Given the low statistical power of the *Q* test with a small number of studies, and because the random-effects model yields more conservative estimates in the presence of heterogeneity while converging to a fixed-effect model in its absence, we adopted a random-effects model for all meta-analyses. The between-study variance (*τ*^2^) was estimated using the DerSimonian–Laird method. If substantial heterogeneity (*I*^2^ > 75%) was detected, meta-analysis was deemed inappropriate, and the results were summarized descriptively (30). A leave-one-out sensitivity analysis was performed to evaluate the robustness of the pooled results by examining the influence of sequentially excluding each study. For outcomes with 10 or more included studies, potential publication bias was assessed through visual inspection of funnel plot symmetry and Egger’s test. To explore the impact of intervention duration on the efficacy of feeding strategies, subgroup analyses were stratified by the duration of enteral nutrition (<7 days versus ≥7 days). Additionally, for the gastric residual volume outcome, a further stratified subgroup analysis was conducted based on variations in diagnostic thresholds (≥250 mL versus <250 mL). For all the analyses, *p* < 0.05 was considered statistically significant.

## 3 Results

### 3.1 Study characteristics

**Figure 1:**
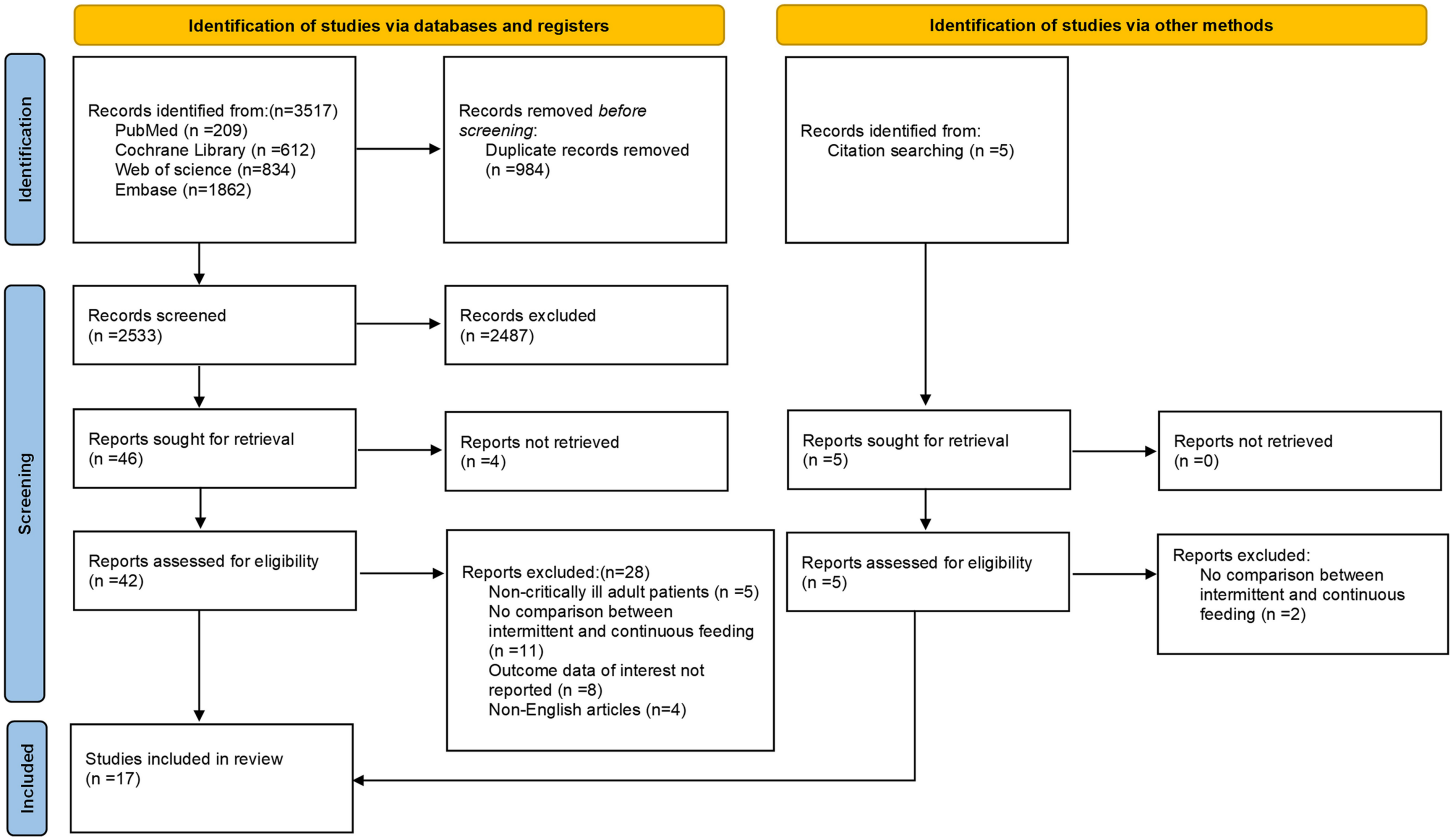
PRISMA flow chart of the study selection process.

Through a systematic database search, 3,517 studies were initially identified. An additional five studies were discovered via citation searching. After removing duplicates, the titles and abstracts of the remaining records were screened for relevance. Subsequently, the full texts of potentially eligible studies were reviewed according to the predefined inclusion and exclusion criteria. Finally, 17 studies (16–19, 31–43) were included in the systematic review, and the detailed literature screening process is illustrated in Figure 1.

The basic characteristics of all included studies are summarized in Table 1, with detailed intervention protocols and primary study results provided in Supplementary Table 2. Analysis of these baseline characteristics revealed the following key findings: The pooled patient population comprised 1,505 individuals, with nutritional regimens stratified into continuous feeding (*n* = 754) and intermittent feeding (*n* = 751) cohorts. All patients were admitted to the ICU. Most included studies implemented standardized nutritional regimens targeting caloric intakes of 25–30 kcal/kg/day. The study durations ranged from 3 to 21 days. The included studies were characterized by relatively small sample sizes, ranging from 18 to 294 participants. It should be noted that the definitions of gastrointestinal tolerance outcomes varied slightly across studies. For example, the high gastric residual volume threshold ranged from 60 to 250 mL, and the criteria for diagnosing diarrhea and constipation were not fully consistent (see Supplementary Table 3). Methodologically, two studies (39, 43) implemented sequential intermittent feeding strategies. Given their shared core feature of non-24-h continuous infusion, sequential intermittent feeding and bolus feeding were uniformly classified as intermittent feeding.

**Table 1:** Characteristics of included studies.

First author/year	Country, study period	Patient population	Sample size (C/I)	Age, years	Sex (M/F)	Caloric goal	Continuous enteral feeding	Intermittent enteral feeding
Bonten (31) 1996	Netherlands, 14 days	Three ICUs (two mixed and one cardiosurgical ICU)	30/30	C: 65 (55/70) I: 68 (61/73)	C: 19/11 I: 16/14	ND	24 h/day	18 h/day, stopping from 2 a.m. to 8 a.m.
Steevens (32) 2002	USA, 7 days	Surgical and medical intensive care units, multiple trauma patients	9/9	C: 37.3 ± 16.5 I: 35.9 ± 14.3	C: 7/2 I: 5/4	25–30 kcal/kg/day	24 h/day, initiated at 25 mL/h and advanced every 12 hours by 25 mL/h until goal rate was achieved	Started with a bolus of 125 mL by force of gravity every 4 h and increased every 12 hours by 125 mL until the goal volume was achieved
Serpa (33) 2003	Brazil, 3 days	Intensive care unit	14/14	C: 69.6 ± 13.2 I: 64.9 ± 13.9	C: 9/5 I: 7/7	25 kcal/kg/day	24 h/day	Administered 8 doses at 3-hour intervals, each infused over 1 hour
Chen (34) 2006	Taiwan China, 7 days	Two intensive care units	51/56	Total: <45: 6.5% 45–64: 20.6% 65–74: 16.8% 75–84: 43.0% >84: 13.1%	C: 39/12 I: 43/13	25 kcal/kg/day	24 h/day	Intermittent feeding 4 to 6 times a day, each with a volume of less than 350 mL, and infused via nasogastric tube in a bolus forced by gravity, within 15–20 min
MacLeod (35) 2007	USA, 7 days	Trauma intensive care unit	81/79	C: 48.4 ± 2.3 I: 44.6 ± 2.3	C: 53/28 I: 60/19	25 kcal/kg/day	24 h/day; initiated at 20 mL/h, increased by 20 mL/h every 8 h.	Initially, feed 100 mL every 4 hours. If the patient tolerates it, increase by 100 mL every 8 hours
Kadamani (18) 2014	Lebanon, 5 days	Intensive care unit	15/15	C: 64.73 ± 16.65 I: 61.60 ± 17.82	C: 9/6 I: 10/5	ND	24 h/day	Administration every 4 to 6 hours, with each session lasting 10–15 min
Tavares de Araujo (36) 2014	Brazil, 5 days	Intensive care unit	23/18	C: 61.3 ± 20.8 I: 68.9 ± 19.4	C: 14/9 I: 10/8	25–30 kcal/kg/day	24 h/day	Feeding for 18 h, with a 6 h nocturnal pause
Mazaherpur (37) 2016	Iran, 21 days	Intensive care unit, traumatic brain injury patients	20/20	C: 32 ± 8.7 I: 34.2 ± 11.3	C: 7/13 I: 4/16	Harris-Benedict formula	18 h/day (6 a.m.–12 p.m.), initiated at 20 mL/h	Started with 50 mL every 3 h
McNelly (16) 2020	United Kingdom, 10 days	Eight mixed intensive care units	59/62	C: 60.3 (56.0–64.1) I: 55.2 (51.0–59.3)	C: 40/19 I: 41/21	Modified Penn State equation or 25 kcal/kg	24 h/day	Administered 6 times daily at 4-hour intervals via nasogastric tube as a gravity-driven bolus over 3–5 min
Rana (38) 2021	India, 3 days	Intensive care unit	30/30	Total: 20–39: 21.7% 40–59: 35% 60–80: 43.3%	C: 23/7 I: 21/9	ND	Initial rate 20–50 mL/h with 10–25 mL increments every 4–24 h	Gravity drip via nasogastric tube using a syringe with a volume of 150–250 mL per defined time period
Ren (39) 2021	Qingdao China, 7 days	Intensive care unit	30/32	C: 55 (48–67) I: 66 (54–72)	C: 19/11 I: 17/15	25–30 kcal/kg/day	24 h/day	Administer three times daily (7–9 a.m., 11 a.m.–1 p.m., 5–7 p.m.) via pump over 2 hours each
Lee (17) 2022	South Korea, 7 days	Medical intensive care unit	50/49	C: 67.5 ± 10.3 I: 66.2 ± 12.7	C: 33/17 I: 33/16	ND	24 h/day, initiated at 25 mL/h	Four times daily at 9 a.m., 1 p.m., 5 p.m., and 9 p.m., each infusion within 1 hour
Banaei (19) 2022	Iran, 7 days	Intensive care unit, trauma patients	31/25	C: 60.16 ± 23.61 I: 69.16 ± 19.57	C: 20/11 I: 13/12	Harris-Benedict formula	24 h/day, initiated at 50 mL/h, increased by 50 mL every 6 h until target	300 mL bolus via 50 mL syringe over 10–20 min, every 3 h
Wilkinson (40) 2023	United Kingdom, 10 days	Eight intensive care units	35/40	C: 61 (55–67) I: 55 (50–60)	C: 9/26 I: 11/29	ND	24 h/day	Administer 6 times daily via nasogastric tube over 3–5 min per feeding
Panwar (41) 2024	Australia, 14 days	Five multidisciplinary intensive care units	59/61	C: 64.8 (50.7–74.8) I: 65.1 (53.2–73.4)	C: 31/28 I: 41/20	25 kcal/kg/day	24 h/day	Three daily feedings (5–6 a.m., 12–1 p.m., and 8–9 p.m.), each delivering one-third of the daily target volume over 30–60 min
Hrdy (42) 2025	Czech Republic, 5 days	Four intensive care units	148/146	C: 64.4 ± 10.9 I: 62.9 ± 13.6	C: 109/39 I: 104/42	25–30 kcal/kg if BMI < 30 kg/m^2^; 11–14 kcal/kg if BMI 30–50 kg/m^2^	18 h/day, initiated at 25 mL/h	The enteral nutrition dose is divided into 6 portions administered every 4 h
Yao (43) 2025	Qingdao China, 7 days	Intensive care unit	69/65	C: 65 (54–75) I: 64 (50–74)	C: 55/14 I: 45/20	25–30 kcal/kg	24 h/day	Three times daily (7–9 a.m., 11 a.m.–1 p.m., 5–7 p.m.), each infusion within 2 h

C, continuous enteral feeding; I, intermittent enteral feeding; ICU, intensive care unit; BMI, body mass index; ND, not described.

### 3.2 Risk of bias assessment

**Figure 2:**
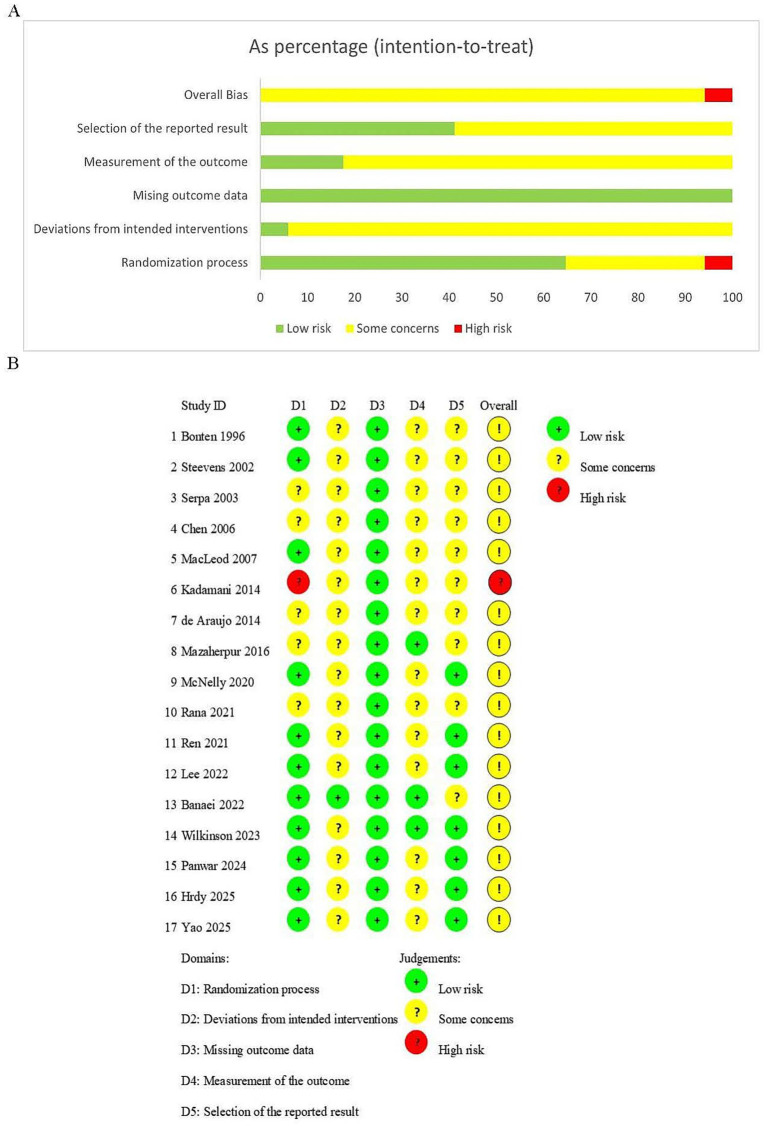
Summary of risk-of-bias assessment: **(A)** Summary of domains for included studies; **(B)** Risk-of-bias summary for each included study.

The results of the risk of bias assessment are summarized in Figure 2. Among the 17 included studies, 16 were rated as having some concerns, and one was rated as high risk. Specifically, regarding the randomization process, five studies mentioned randomization but did not describe allocation concealment and were rated as having some concerns; one study was rated as high risk due to its use of a quasi-randomized design. Due to the inherent constraints of enteral nutrition interventions, blinding was difficult to implement. Only one study, which employed triple blinding, was rated as low risk. The remaining 16 studies were classified as having some concerns owing to inadequate reporting on blinding implementation. For outcome measurement, 14 studies were rated as having some concerns owing to unconfirmed blinding or unclear objectivity of the measurement methods. Regarding selective reporting, 10 studies were rated as having some concerns due to the absence of prospective registration or failure to distinguish between prespecified and exploratory analyses.

### 3.3 Heterogeneity

Owing to substantial heterogeneity in outcome assessments (*I*^2^ > 75%), a meta-analysis was not feasible for all outcomes. Therefore, descriptive summaries were provided for outcomes such as pneumonia and achieved energy intake, while the remaining outcome data were pooled for analysis.

### 3.4 Results of the study: primary outcomes

#### 3.4.1 Diarrhea

**Figure 3:**
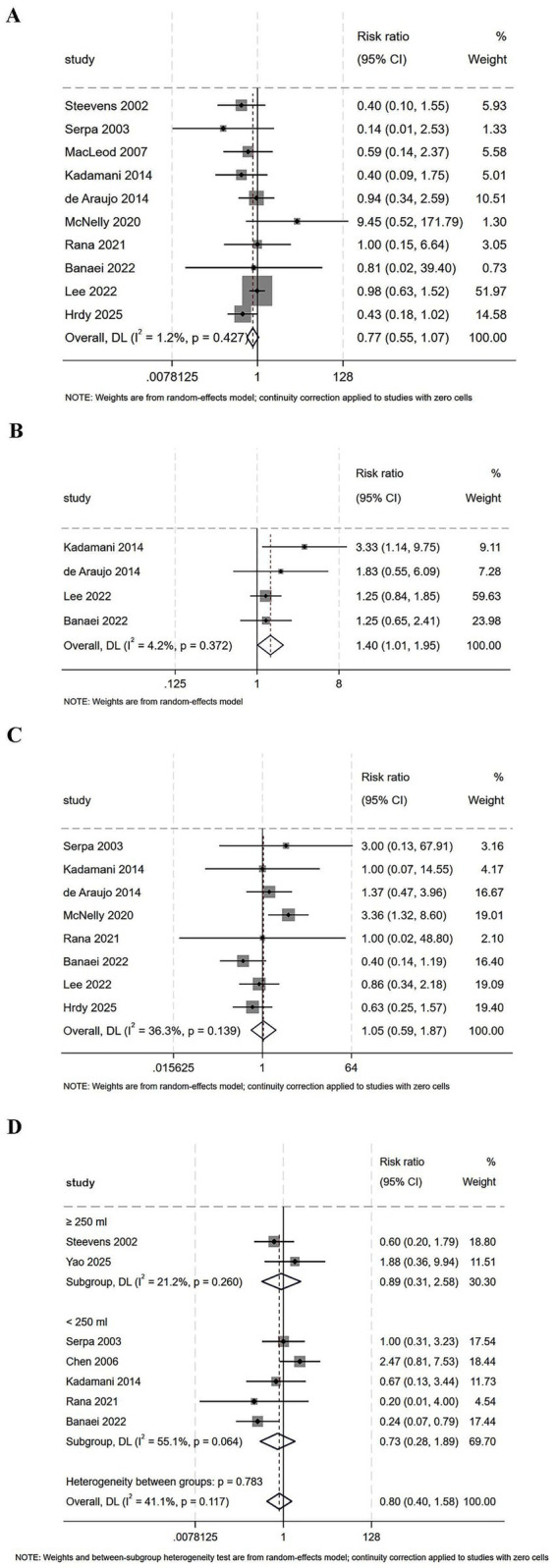
Forest plot comparing continuous versus intermittent enteral feeding for the outcomes of **(A)** diarrhea, **(B)** constipation, **(C)** vomiting, and **(D)** gastric residual volume.

Ten studies compared the risk of diarrhea between continuous and intermittent enteral feeding. The pooled result demonstrated that the risk of diarrhea was not significantly different between the two groups (RR = 0.77, 95% CI = 0.55–1.07, *I*^2^ = 1.2%, Figure 3A).

#### 3.4.2 Constipation

Four studies assessed the risk of constipation between continuous and intermittent enteral feeding. The pooled result indicated that continuous feeding was associated with an increased risk of constipation relative to intermittent feeding (RR = 1.40, 95% CI = 1.01–1.95, *I*^2^ = 4.2%, Figure 3B).

#### 3.4.3 Vomiting

Eight studies investigated the risk of vomiting between continuous and intermittent enteral feeding. The pooled result demonstrated no significant difference in the risk of vomiting between the two groups (RR = 1.05, 95% CI = 0.59–1.87, *I*^2^ = 36.3%, Figure 3C).

#### 3.4.4 Gastric residual volume

Seven studies compared the risk of high gastric residual volume between continuous and intermittent enteral feeding. The pooled result showed that the gastric residual volume was not significantly different between the two groups (RR = 0.80, 95% CI = 0.40–1.58, *I*^2^ = 41.1%, Figure 3D). Subgroup analyses revealed no significant differences in this risk between the two groups, both in the ≥250 mL subgroup (RR = 0.89, 95% CI = 0.31–2.58, *I*^2^ = 21.2%, Figure 3D) and in the <250 mL subgroup (RR = 0.73, 95% CI = 0.28–1.89, *I*^2^ = 55.1%, Figure 3D).

#### 3.4.5 Abdominal distension

**Figure 4:**
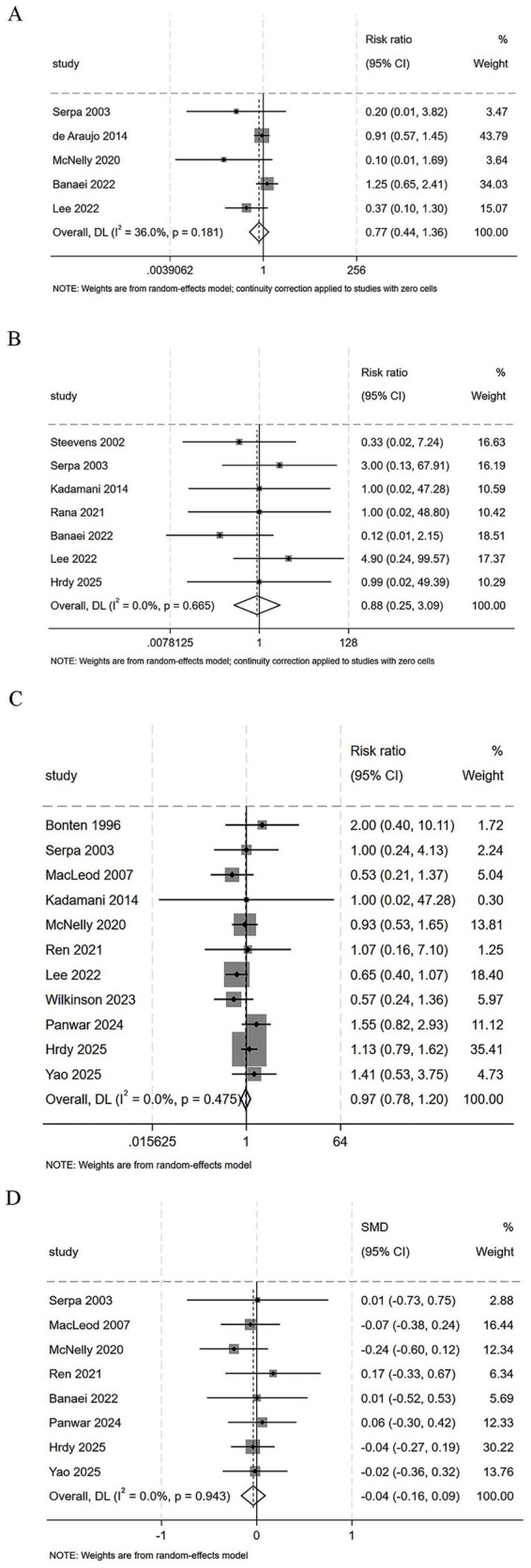
Forest plot comparing continuous versus intermittent enteral feeding for the outcomes of **(A)** abdominal distension, **(B)** aspiration, **(C)** ICU mortality, and **(D)** length of ICU stay.

Five studies assessed the risk of abdominal distension between continuous and intermittent enteral feeding. The pooled result demonstrated that the risk of abdominal distension was not significantly different between the two groups (RR = 0.77, 95% CI = 0.44–1.36, *I*^2^ = 36.0%, Figure 4A).

#### 3.4.6 Aspiration

Seven studies compared the risk of aspiration between continuous and intermittent enteral feeding. The pooled result indicated that the risk of aspiration was not significantly different between the two groups (RR = 0.88, 95% CI = 0.25–3.09, *I*^2^ = 0.0%, Figure 4B).

#### 3.4.7 Pneumonia

Four studies investigated the risk of pneumonia between continuous and intermittent enteral feeding. Owing to considerable heterogeneity across these studies (*I*^2^ = 78.6%), meta-analysis was deemed inappropriate. Three studies (31, 35, 42) reported no statistically significant difference in pneumonia risk between feeding regimens. Conversely, Chen et al. (34) reported a lower risk of pneumonia with intermittent enteral feeding. In summary, the available evidence is insufficient to confirm a definitive effect of the two feeding regimens on pneumonia risk.

### 3.5 Results of the study: secondary outcomes

#### 3.5.1 ICU mortality

Eleven studies assessed ICU mortality between continuous and intermittent enteral feeding. The pooled result showed that ICU mortality was not significantly different between the two groups (RR = 0.97, 95% CI = 0.78–1.20, *I*^2^ = 0.0%, Figure 4C).

#### 3.5.2 Length of ICU stay

Eight studies compared ICU length of stay between continuous and intermittent enteral feeding. The pooled result showed that the length of ICU stay was comparable between the two groups (SMD = −0.04, 95% CI = −0.16 to 0.09, *I*^2^ = 0.0%, Figure 4D).

#### 3.5.3 Achieved energy intake

Three studies reported achieved energy intake for continuous versus intermittent enteral feeding. Due to substantial heterogeneity among the included studies (*I*^2^ = 85.2%), a meta-analysis was not performed. Two studies (16, 35) demonstrated that intermittent enteral feeding was associated with faster achievement of the energy intake target, whereas the remaining study (37) found no statistically significant difference between the two feeding regimens. Synthesizing the available evidence, intermittent enteral feeding may offer a potential advantage over continuous enteral feeding in achieving the target energy intake. However, given the limited number of studies and substantial heterogeneity, this finding warrants further validation.

### 3.6 Sensitivity analysis and publication bias

A leave-one-out sensitivity analysis was performed for all outcomes. The results indicated that the pooled effect estimates were robust across all outcomes (see Supplementary Figures 1, 2). For outcomes with 10 or more included studies (diarrhea and ICU mortality), funnel plots exhibited no significant asymmetry (see Supplementary Figure 3), and Egger’s test confirmed the absence of significant publication bias for diarrhea (*p* = 0.517) and ICU mortality (*p* = 0.976).

### 3.7 Subgroup analysis

To further examine the impact of enteral feeding intervention duration on outcomes, subgroup analyses were conducted by stratifying the intervention duration into <7 days and ≥7 days. In the <7 days intervention subgroup, continuous feeding was linked to an elevated risk of constipation (RR = 2.55, 95% CI = 1.15–5.69, *I*^2^ = 0.0%). In contrast, no significant difference was observed in the subgroup with intervention duration ≥7 days (RR = 1.25, 95% CI = 0.89–1.75, *I*^2^ = 0.0%). For all other outcomes, including diarrhea, vomiting, gastric residual volume, abdominal distension, aspiration, ICU mortality, and ICU length of stay, no significant differences were observed between the two feeding regimens in either duration-based subgroup. The detailed results of the subgroup analyses are presented in Supplementary Table 4.

### 3.8 Certainty of evidence

Using the GRADE approach for systematic assessment of evidence quality, the synthesized evidence was rated as low or very low (see Supplementary Table 5). Regarding risk of bias (Domain 1), all studies were judged to have serious limitations primarily due to the inherent challenges in blinding procedures and inadequate reporting of allocation concealment in enteral nutrition studies. No serious concerns were noted for inconsistency or indirectness (Domains 2 and 3). Assessment of imprecision (Domain 4) revealed very serious limitations for most outcomes, characterized by small sample sizes and risk ratios clustered around 1.0. Funnel plots showed no significant asymmetry for any outcome measure, suggesting no obvious publication bias (Domain 5).

## 4 Discussion

This meta-analysis systematically compared the effects of continuous versus intermittent enteral feeding in critically ill patients. The results showed that continuous enteral feeding was associated with an elevated risk of constipation compared with intermittent feeding. No statistically significant differences were observed between the two groups in terms of diarrhea, vomiting, gastric residual volume, abdominal distension, aspiration, ICU mortality, and length of ICU stay. Further subgroup analyses demonstrated that this higher risk was only evident in the subgroup with intervention duration <7 days, whereas this association was not statistically significant in the subgroup with intervention duration ≥7 days.

From a clinical mechanism perspective, the occurrence of constipation may be associated with multiple mechanisms, including gastrointestinal motility disorders (such as delayed gastric emptying and intestinal dysmotility), medication use (for example, sedatives, opioids, and vasopressors), hyperglycemia, and electrolyte disturbances (44). Research suggests that intermittent feeding promotes the pulsatile release of gastrointestinal hormones. This includes stimulating cholecystokinin (CCK) to enhance gallbladder emptying and maintaining incretin levels such as glucagon-like peptide-1 (GLP-1) and glucose-dependent insulinotropic polypeptide (GIP), thereby coordinating carbohydrate metabolism and muscle protein synthesis (45). Our core finding that continuous enteral feeding elevates constipation risk corroborates the report by Heffernan et al. (21) and further indicates a concentration of this risk in the subgroup with intervention duration <7 days. This may be attributed to the gastrointestinal function of critically ill patients not having yet adapted to nutritional support in the initial stage. During this period, intermittent feeding, by mimicking natural eating cycles, may help maintain intestinal motility rhythms. In contrast, the lack of such physiological intervals in continuous feeding may suppress the periodic regulation of intestinal motility, thereby increasing the constipation risk in the subgroup with intervention duration <7 days.

Gastric residual volume is an indicator used to assess gastric emptying and enteral feeding tolerance. This study found no significant difference between the two enteral feeding regimens in this measure. However, a meta-analysis by Ma et al. (20) indicated that intermittent feeding might increase gastric residual volume during long-term enteral nutrition (beyond 1 week). Previous studies often combined aspiration and aspiration pneumonia as a single outcome measure or focused exclusively on one of them. In contrast, this study evaluated pneumonia and aspiration as distinct and independent endpoints. Although aspiration during enteral nutrition is a significant risk factor for the development of pneumonia, the two should not be conflated. Unfortunately, the analysis of pneumonia risk in this study demonstrated considerable heterogeneity, which could be attributed to the limited number of included studies. The rapid administration of formula during intermittent feeding can lead to a sudden rise in gastric pressure, thereby predisposing patients to aspiration, nausea, and vomiting (46). A meta-analysis by Tong et al. (22) indicated that continuous enteral feeding, as opposed to intermittent enteral feeding, was associated with an elevated risk of vomiting. The underlying mechanisms remain incompletely elucidated. It is plausible that continuous feeding contributes to gastroesophageal reflux by prolonging periods of patient immobility. Alternatively, it may disrupt circadian rhythms, thereby adversely affecting gastrointestinal motility and function (13). Additionally, a meta-analysis by Qu et al. (23) suggested that continuous enteral feeding may be associated with an increased risk of diarrhea and abdominal distension. Enteral feeding itself may indirectly contribute to gut microbiota dysbiosis through mechanisms such as altered intestinal barrier function, enhanced oxidative stress, or promotion of pathogenic bacterial overgrowth (47). Furthermore, during continuous enteral feeding, especially with overnight infusion, inadequate temperature maintenance of the nutrient solution may occur. These temperature fluctuations could represent a potential contributing factor to diarrhea.

Regarding secondary outcomes, although intermittent feeding offers advantages such as operational flexibility and potential recovery benefits, this study did not find a significant reduction in patient mortality with this approach. However, a meta-analysis by Wu et al. (24) indicated that continuous enteral feeding could contribute to a reduction in mortality among ICU patients. The current findings regarding mortality lack robust supporting evidence, which may be attributed to the short follow-up durations in the included studies, generally ranging from 3 to 7 days. As mortality is a long-term endpoint, it cannot be reliably assessed within such a brief observation period. Although short-term follow-up precludes assessment of long-term survival, it nonetheless provides an essential window for observing early enteral nutrition-related feeding intolerance, including diarrhea, constipation, and vomiting. A study (48) reported that approximately 24% of ICU patients experienced at least one episode of enteral feeding intolerance, with a daily incidence that was about 1% on the first ICU day, peaked at 6% within 4–5 days after admission, and subsequently declined. This pattern highlights the prominence of feeding intolerance during the early phase of enteral nutrition. Furthermore, several studies suggest that intermittent feeding may facilitate better achievement of target energy intake. Animal studies have shown that piglets receiving intermittent enteral feeding exhibit twice the protein synthesis rate of those on continuous enteral feeding regimens, indicating that this feeding pattern may stimulate greater muscle protein accretion (49). Negative nitrogen balance is common in critically ill patients. A study (50) found that giving amino acids through intermittent feeding changed the body’s protein balance from negative to positive. Similarly, intermittent enteral feeding is considered more advantageous than continuous enteral feeding in terms of promoting protein synthesis and improving nutritional intake.

Current guidelines from the American Society for Parenteral and Enteral Nutrition (ASPEN) and the European Society for Clinical Nutrition and Metabolism (ESPEN) recommend continuous enteral feeding as the primary nutritional strategy for critically ill patients, with an emphasis on ensuring adequate nutrient intake (3, 51). In contrast, the present study specifically focused on gastrointestinal tolerance and demonstrated that continuous enteral feeding was associated with an elevated risk of constipation, which was particularly pronounced among patients with an intervention duration <7 days. These findings suggest that for patients at high risk of constipation, especially those anticipated to require short-term enteral nutrition, intermittent feeding may offer a beneficial and individualized alternative. However, the broader clinical benefits of intermittent enteral feeding in improving other clinical outcomes require further validation. Notably, no significant differences were observed between the two feeding regimens regarding the majority of other clinical outcomes. Therefore, the selection of an enteral feeding regimen should be based on a comprehensive assessment of individual patient characteristics, including disease severity, gastrointestinal function, anticipated feeding duration, and the availability of institutional resources, to provide personalized nutritional support.

## 5 Limitation

This meta-analysis has several limitations. First, due to the nature of enteral feeding interventions, clinical staff could easily distinguish between comparison groups. Most of the included studies did not implement blinding or allocation concealment, which may have reduced the methodological quality of the evidence. Second, the sample sizes of the included studies were relatively small (all <100), and the intervention durations were short (all <21 days), likely reflecting constraints typical of intensive care settings. Consequently, the statistical power to evaluate long-term endpoints such as mortality is likely insufficient, with the relevant conclusions requiring cautious interpretation. Third, inevitable variations exist across the included studies in the implementation protocols of intermittent enteral feeding and the criteria for outcome assessment. These variations represent a potential source of heterogeneity and may thus moderately limit the generalizability of the synthesized findings. Finally, the restriction to English-language publications may have introduced selection bias, thereby reducing the external validity of our findings across diverse healthcare settings.

## 6 Conclusion

In conclusion, current evidence indicates that continuous enteral feeding is associated with a higher risk of constipation in critically ill patients, with this effect being particularly pronounced in patients with an intervention duration <7 days. For other outcomes, no statistically significant differences were observed between the two feeding regimens. Nevertheless, these conclusions should be interpreted with caution due to limitations including the overall low quality of evidence and small sample sizes in the included studies. Consequently, in clinical practice, there is no universally “optimal” enteral feeding strategy; instead, individualized feeding plans should be developed based on a comprehensive assessment of individual patient factors. Future large-scale, high-quality studies with long-term follow-up are necessary to further validate the efficacy of intermittent enteral feeding in alleviating gastrointestinal intolerance.

## Data Availability

The original contributions presented in the study are included in the article/Supplementary material, further inquiries can be directed to the corresponding author.
