# Supplementary material for: Effects of continuous versus intermittent enteral feeding on feeding tolerance in critically ill adults: a systematic review and meta-analysis
Source: Front Med (Lausanne). 2026 Jan 21;13:1738036. doi: 10.3389/fmed.2026.1738036 (PMC12868274; doi:10.3389/fmed.2026.1738036)
Supplement: Supplementary file 1 [file Data_Sheet_1.docx]

**Supplementary Material**

**Supplementary Table 1. Search strategy**

| Database | No. | Query | Results |
| --- | --- | --- | --- |
| PubMed | #1 | (enteral nutrition[MeSH Terms] OR enteral nutrition[Title/Abstract] OR tube feed*[Title/Abstract] OR enteral feed*[Title/Abstract] OR gastric feed*[Title/Abstract] OR nutrition* support[Title/Abstract] OR nutrition* therap*[Title/Abstract] OR enteral[Title/Abstract] OR feed*[Title/Abstract] OR nutrition[Title/Abstract] OR enteral formula*[Title/Abstract] OR artificial nutrition[Title/Abstract] OR feeding regimen*[Title/Abstract] OR nasogastric tube[Title/Abstract]) | 866183 |
|  | #2 | (continuous[Title/Abstract] OR continuously[Title/Abstract] OR intermittent[Title/Abstract] OR bolus[Title/Abstract] OR discontinuous[Title/Abstract] OR sequential[Title/Abstract] OR continuous infusion[Title/Abstract] OR intermittent bolus[Title/Abstract] OR sequential feeding[Title/Abstract] OR gravity feeding[Title/Abstract] OR pump feeding[Title/Abstract] OR syringe feeding[Title/Abstract] OR administration method*[Title/Abstract] OR delivery method*[Title/Abstract]) | 1008395 |
|  | #3 | (intensive care units[MeSH Terms] OR critical care[MeSH Terms] OR critical illness[MeSH Terms] OR intensive care units[Title/Abstract] OR ICU[Title/Abstract] OR ICUs[Title/Abstract] OR critical care[Title/Abstract] OR critical illness[Title/Abstract] OR critical patients[Title/Abstract] OR mechanical ventilation[Title/Abstract] OR critical care unit[Title/Abstract]) | 331348 |
|  | #4 | (adult[MeSH Terms] OR adult[Title/Abstract] OR adults[Title/Abstract] OR aged[MeSH Terms] OR aged[Title/Abstract] OR elderly[Title/Abstract] OR adult patients[Title/Abstract]) | 9793496 |
|  | #5 | (randomized controlled trial[Publication Type] OR clinical trial[Publication Type] OR randomized controlled trial[Title/Abstract] OR clinical trial[Title/Abstract] OR RCT[Title/Abstract] OR RCTs[Title/Abstract] OR randomized[Title/Abstract]) | 1571285 |
|  | #6 | #1 AND #2 AND #3 AND #4 AND #5 | 209 |
| Cochrane | #1 | MeSH descriptor: [Enteral Nutrition] explode all trees | 2481 |
|  | #2 | MeSH descriptor: [Nutrition Therapy] explode all trees | 12907 |
|  | #3 | MeSH descriptor: [Nutritional Support] explode all trees | 4452 |
|  | #4 | (enteral nutrition OR nutrition therapy OR nutritional support OR tube feed* OR enteral feed* OR gastric feed* OR enteral OR feed* OR nutrition OR enteral formula* OR artificial nutrition OR feeding regimen*):ti,ab,kw | 148349 |
|  | #5 | #1 OR #2 OR #3 OR #4 | 155048 |
|  | #6 | (continuous OR continuously OR intermittent OR bolus OR discontinuous OR sequential OR intermittent bolus OR sequential feeding OR gravity feeding OR pump feeding OR syringe feeding):ti,ab,kw | 136223 |
|  | #7 | MeSH descriptor: [Intensive Care Units] explode all trees | 6304 |
|  | #8 | MeSH descriptor: [Critical Care] explode all trees | 3060 |
|  | #9 | MeSH descriptor: [Critical Illness] explode all trees | 3821 |
|  | #10 | MeSH descriptor: [Respiration, Artificial] explode all trees | 9408 |
|  | #11 | (intensive care units OR mechanical ventilation OR critical care OR critical illness OR critical patients OR critically ill OR severe illness):ti,ab,kw | 64051 |
|  | #12 | #7 OR #8 OR #9 OR #10 OR #11 | 68078 |
|  | #13 | MeSH descriptor: [Adult] explode all trees | 627217 |
|  | #14 | MeSH descriptor: [Aged] explode all trees | 283246 |
|  | #15 | (adult OR aged OR elderly):ti,ab,kw | 1148038 |
|  | #16 | #13 OR #14 OR #15 | 1148468 |
|  | #17 | (randomized controlled trial OR clinical trial OR RCT OR RCTs OR randomized):ti,ab,kw | 1378035 |
|  | #18 | #5 AND #6 AND #12 AND #16 AND #17 | 612 |
| Web of Science | #1 | TS=(enteral nutrition OR tube feed* OR enteral feed* OR gastric feed* OR nutrition* support OR nutrition* therap* OR enteral OR feed* OR nutrition OR enteral formula* OR artificial nutrition OR feeding regimen*) | [1943514](https://www.webofscience.com/wos/woscc/summary/e01d8033-7e47-449c-812d-14022a8e48d2-01858f8930/relevance/1) |
|  | #2 | TS=(continuous feed* OR intermittent feed* OR bolus feed* OR continuous infusion OR intermittent bolus OR cyclic feed* OR sequential feed* OR gravity feed* OR pump feed* OR syringe feed* OR administration method* OR delivery method* OR infusion rate OR feed* regimen* OR feed* rate) | [1114401](https://www.webofscience.com/wos/woscc/summary/6868a8f1-fca3-400f-a9d2-ea68b764ea36-01858f90b9/relevance/1) |
|  | #3 | TS=(intensive care units OR critical care OR critical illness OR ICU OR critical patients OR critically ill OR severe illness OR mechanical ventilation OR intensive care OR critical care unit*) | [779110](https://www.webofscience.com/wos/woscc/summary/27b21931-f5d6-4317-8ac9-7be974256d70-01858f9535/relevance/1) |
|  | #4 | TS=(adult OR adults OR aged OR elderly OR adult patients) | [6353172](https://www.webofscience.com/wos/woscc/summary/d68b3cff-d8d1-4c12-83be-50490eaa8c9b-01858f9880/relevance/1) |
|  | #5 | TS=(randomized controlled trial OR clinical trial OR RCT OR RCTs OR randomized) | [1826473](https://www.webofscience.com/wos/woscc/summary/cd7128d3-c758-4fb7-aa3d-4b340f020f14-01858f9ba2/relevance/1) |
|  | #6 | #1 AND #2 AND #3 AND #4 AND #5 | [834](https://webofscience.clarivate.cn/wos/woscc/summary/82a6560e-ab87-4e5c-9bd1-9a2a0c17ce45-016e5707b4/relevance/1) |
| Embase | #1 | 'enteric feeding'/exp OR 'nutrition supplement'/exp OR 'feeding tube'/exp OR 'feeding'/exp OR 'nutrition'/exp OR 'artificial feeding'/exp OR 'enteral feeding pump '/exp OR 'feeding tube':ab,ti OR 'enteric feeding':ab,ti OR 'nutrition supplement':ab,ti OR 'diet therapy':ab,ti OR 'feeding':ab,ti | [3357115](http://www--embase--com--https.embase.shd1rmyy.lwnote.com:50001/) |
|  | #2 | 'continuous infusion'/exp OR continuous:ab,ti OR continuously:ab,ti OR intermittent:ab,ti OR 'bolus':ab,ti OR discontinuous:ab,ti OR sequential:ab,ti OR 'continuous infusion':ab,ti OR 'intermittent bolus':ab,ti OR 'gravity feeding':ab,ti OR 'syringe feeding':ab,ti | 1409034 |
|  | #3 | 'intensive care unit'/exp OR 'intensive care'/exp OR 'critically ill patient'/exp OR 'artificial ventilation'/exp OR 'critical illness':ab,ti OR 'critical patients':ab,ti OR 'critically ill patient':ab,ti OR 'intensive care unit':ab,ti OR 'severe illness':ab,ti OR icu:ab,ti | 1382897 |
|  | #4 | 'adult'/exp OR 'aged'/exp OR adults:ab,ti OR 'adult':ab,ti OR 'aged':ab,ti OR elderly:ab,ti | 14389086 |
|  | #5 | 'randomized controlled trial'/exp OR 'controlled clinical trial'/exp OR 'randomized controlled trial':ab,ti OR 'controlled clinical trial':ab,ti OR 'clinical trial':ab,ti OR rct:ab,ti OR rcts:ab,ti | 1668281 |
|  | #6 | #1 AND #2 AND #3 AND #4 AND #5 | 1862 |

**Supplementary Table 2. Enteral Nutrition Intervention Regimen and Key Findings by Study**

| First author/year | Continuous Feeding Group | Intermittent/Bolus/Sequential  Feeding Group | Key finding | |
| --- | --- | --- | --- | --- |
| Bonten (31)  1996 | Continuous enteral feeding was administered continuously over 24 hours via an electric infusion pump at a constant rate without interruption. | Intermittent enteral feeding was scheduled as a continuous infusion via an electric infusion pump over an 18-hour period each day, with administration discontinued between 2 a.m. to 8 a.m. This interval was selected for practical reasons. In each patient, enteral feeding was initiated at 6 p.m. according to the following volume‑escalation protocol: 500 mL on the first day, 1,000 mL on the second day, 1,500 mL on the third day, and 2,000 mL from the fourth day onward. | Pneumonia and ICU mortality rates were similar between continuous and intermittent feeding groups. | |
| Steevens (32)  2002 | Continuous enteral feeding was initiated at 25 mL/h and advanced by 25 mL/h every 12 hours until the goal infusion rate for each patient was achieved. | Bolus enteral feeding was initiated with 125 mL delivered by gravity over a 15-minute period every 4 hours. The volume was subsequently advanced by 125 mL every 12 hours until the target feeding volume was attained. | Continuous feeding was associated with fewer gastrointestinal complications compared to intermittent feeding. | |
| Serpa (33)  2003 | Continuous enteral feeding was delivered via an electronic infusion pump, providing the total daily volume over a continuous 24-hour period. | Intermittent enteral feeding was delivered over a total daily period of 24 hours using an electronic infusion pump. The regimen consisted of eight equal aliquots, each administered over a 1-hour period at 3-hour intervals (each 1-hour infusion followed by a 2-hour standby period). | No significant difference was observed between continuous and intermittent feeding regimens. | |
| Chen (34)  2006 | Continuous enteral feeding was administered at a constant rate over a 24-hour period using a feeding pump. On Day 1, a 5% glucose solution was infused at a rate of 25 mL/h, resulting in a total volume of 500 mL. From Day 2 onward, enteral formula was provided according to the established energy requirements. | Bolus enteral feeding was administered 4 to 6 times daily, with each bolus volume limited to < 350 mL. Boluses were delivered by ICU nurses via gravity over 15-20 minutes. On Day 1, 125 mL of 5% dextrose was given every 4 hours, yielding a total volume of 500 mL. From Day 2 onward, enteral formula was provided according to the predetermined energy requirements. | No difference in gastric residual volume was observed between continuous and intermittent feeding; however, intermittent feeding was associated with a lower risk of pneumonia. | |
| MacLeod (35)  2007 | Continuous enteral feeding was delivered via a feeding pump. The regimen commenced at 20 mL/h for 8 hours and was advanced by 20 mL/h every 8 hours thereafter until the target volume was reached. | Intermittent enteral feeding was initiated with 100 mL administered every 4 hours for two consecutive cycles. If tolerated, the volume was escalated by 100 mL every 8 hours until the target volume (defined as one‑sixth of the 24-hour nutritional goal) was attained. Each feeding was delivered via an enteral infusion pump over 30-60 minutes. | No statistically significant difference was found in intestinal complications between continuous and intermittent feeding; however, patients receiving intermittent feeding achieved nutritional targets more rapidly. | |
| Kadamani (18)  2014 | Continuous enteral feeding was provided via an enteral feeding pump over a 24-hour period without interruption. | Bolus enteral feeding was delivered by gravity using a Tommy syringe, with each administration completed within 10-15 minutes and repeated every 4-6 hours. | Continuous versus intermittent feeding did not affect the incidence of aspiration, high gastric residual volume, vomiting, or diarrhea. However, patients receiving continuous feeding demonstrated a significantly higher incidence of constipation. | |
| Tavares de Araujo (36)  2014 | Continuous enteral feeding was administered continuously over 24 hours daily via an infusion pump without interruption. | Intermittent enteral feeding was delivered over 18 hours daily via an infusion pump, incorporating a scheduled 6-hour nocturnal pause. | No statistically significant differences were observed between continuous and intermittent enteral feeding groups. | |
| Mazaherpur (37)  2016 | Continuous enteral feeding was administered via a feeding pump over an uninterrupted 24-hour period. The regimen commenced daily at 6 a.m. at an initial rate of 20 mL/h. If tolerated, the infusion rate was advanced by 10-15 mL/h until the target energy requirement was attained. | Intermittent enteral feeding was initiated at a volume of 50 mL every 3 hours. Based on physician assessment and patient tolerance, the feeding volume was escalated progressively to a maximum of 400 mL per administration. | Both continuous and intermittent feeding resulted in energy deficiency; however, the continuous enteral nutrition group demonstrated significantly improved nitrogen balance. | |
| McNelly (16)  2020 | Continuous enteral feeding was administered as a 24-hour infusion of the total daily volume, in accordance with the local standard protocol at each participating center. | Bolus enteral feeding was uniformly implemented across all participating centers. The standardized regimen consisted of six bolus feeds administered at 4-hour intervals over a 24-hour period. Each bolus was delivered via a nasogastric tube using a syringe, with a rapid infusion duration of 3 to 5 minutes per feed. | No differences were observed in safety profiles or gastrointestinal intolerance between continuous and intermittent feeding; however, intermittent feeding was associated with better achievement of nutritional targets. | |
| Rana (38)  2021 | Continuous enteral feeding was delivered using an electrical enteral feeding pump, starting at 20-50 mL/h and advanced by 10-25 mL every 4-24 hours based on tolerance. | Bolus enteral feeding was administered via nasogastric tube using a syringe with gravity drip, with each feed delivering approximately 150-250 mL over a specified time period. | No significant difference was observed in complications between feeding modalities. | |
| Ren (39)  2021 | Continuous enteral feeding was maintained in patients randomly assigned to this group following the attainment of ≥ 80% of the caloric target (25-30 kcal/kg/day) during the initial continuous feeding phase. These patients continued to receive nutrition via an enteral feeding pump at a constant rate over 24 hours daily. | Sequential enteral feeding was implemented in patients randomly allocated to this group after reaching the same nutritional threshold. The total daily enteral nutrition volume was equally distributed across three-time windows: 7-9 a.m., 11 a.m.-1 p.m., 5-7 p.m. Each portion was infused at a constant rate over 2 hours using an enteral feeding pump, with the remaining hours of the day designated as the fasting period. | Continuous and intermittent feeding demonstrated comparable efficacy. | |
| Lee (17)  2022 | Continuous enteral feeding was delivered via nasogastric tube using a feeding pump as a 24-hour continuous infusion. Gastric residual volume was measured every 6 hours with a 50 mL syringe. Starting at 25 mL/h, the infusion rate was adjusted based on residuals and tolerance: if residuals were < 250 mL, the rate was increased by 25 mL/h every 6 hours until the target rate was achieved; if a single residual was ≥ 250 mL, the rate was maintained and metoclopramide (10 mg every 8 h for 48 h) was administered; if two consecutive residuals were ≥ 250 mL, the rate was decreased by 25 mL/h; if three consecutive residuals were ≥ 250 mL, feeding was paused until the next measurement. In case of vomiting, feeding was interrupted and later resumed at 25 mL/h, with metoclopramide considered as needed. | Intermittent enteral feeding was administered via nasogastric tube at 9 a.m., 1 p.m., 5 p.m., and 9 p.m. daily, using gravity infusion to be completed within 1 hour. Gastric residual volume was measured before each feeding with a 50 mL syringe. The initial volume was 150 mL and was adjusted based on residuals and tolerance: if two consecutive residuals were < 250 mL, the volume was advanced until nutritional targets were met; if a single residual was ≥ 250 mL, the volume was maintained and metoclopramide (10 mg every 8 h for 48 h) was given; if two consecutive residuals were ≥ 250 mL, the volume was reduced; if vomiting occurred, feeding was paused and later resumed at 150 mL with metoclopramide. | Continuous feeding demonstrated superior achievement of target nutritional requirements compared to intermittent feeding. | |
| Banaei (19)  2022 | Continuous enteral feeding was administered via a syringe pump as a 24-hour constant‑rate infusion. The regimen started at 50 mL/h and was advanced by 50 mL/h every 6 hours until the target calorie and volume requirements were met. | Bolus enteral feeding was delivered using a 50 mL syringe under gravity, with each 300 mL bolus administered over 10-20 minutes every 3 hours. | Intermittent feeding was associated with increased gastric residual volumes compared to continuous feeding. | |
| Wilkinson (40)  2023 | Continuous enteral feeding was delivered as a 24-hour uninterrupted infusion via an enteral feeding pump, following site-specific clinical protocols. | Bolus enteral feeding was administered as six feeds per 24 hours, each delivered via a nasogastric tube over 3 to 5 minutes (typically using a syringe under gravity). | Continuous and intermittent feeding demonstrated equivalent efficacy. | |
| Panwar (41)  2024 | Continuous enteral feeding was administered via a peristaltic infusion pump over 24 hours without interruption, in accordance with the standard institutional feeding protocols of each participating center. | Intermittent enteral feeding was delivered via a peristaltic infusion pump as three daily bolus feeds, each lasting 30-60 minutes and providing up to one-third of the daily target feed volume (bolus target). The recommended feeding windows were 5-6 a.m., 12-1 p.m., and 8-9 p.m. The initial bolus volume was set at 150 mL, with subsequent volumes increased by 100-150 mL per feed until the target bolus volume was achieved. | Continuous and intermittent feeding demonstrated comparable efficacy. | |
| Hrdy (42)  2025 | Continuous enteral feeding was administered via an enteral feeding pump over 18 hours daily. Throughout the infusion, patients were maintained in a semi-recumbent position at 30-40°. The infusion rate was initiated at 25 mL/h and adjusted stepwise according to enteral tolerance and gastric residual volume. If no signs of intolerance were observed and gastric residual volume remained below 250 mL on two consecutive measurements, the rate was increased by 25 mL every 24 hours until the target rate was reached. If no intolerance was noted and gastric residual volume was between 250-500 mL, the infusion rate remained unchanged. If signs of intolerance emerged or gastric residual volume exceeded 500 mL, the infusion rate was reduced by half until the next tolerance assessment. | Intermittent enteral feeding was implemented by dividing the total daily enteral nutrition volume into six equal portions (boluses), each administered at 4-hour intervals via an enteral feeding pump. The infusion duration and rate for each bolus were adjusted according to its volume: 80 mL and 160 mL boluses were administered over 30 minutes (corresponding to rates of 160 mL/h and 320 mL/h, respectively); a 240 mL bolus was administered over 45 minutes (rate 320 mL/h); 320 mL and 400 mL boluses were administered over 60 minutes (rates 320 mL/h and 400 mL/h, respectively); and boluses exceeding 400 mL were administered at a maximum rate of 400 mL/h until completion. | Intermittent enteral feeding achieved energy targets more rapidly. | |
| Yao (43)  2025 | Continuous enteral feeding was administered to the assigned group after achieving ≥ 80% of the caloric target (25-30 kcal/kg/day). These patients continued on the initial 24-hour continuous infusion protocol, delivered via an enteral feeding pump at a constant rate. | Sequential enteral feeding was initiated in the corresponding group once the same caloric threshold was met. The daily feeding volume was divided equally into three bolus feeds, administered during the following time windows: 7-9 a.m., 11 a.m.-1 p.m., 5-7 p.m. Each bolus was infused at a constant rate over 2 hours using an enteral feeding pump. The other times of the day remained fasting times. | | Continuous and intermittent feeding demonstrated equivalent efficacy. |

Abbreviations: ICU, intensive care unit.

**Supplementary Table 3. Summary of Outcome Measure Definitions in Included Studies**

| First author/year | Outcomes and definition |
| --- | --- |
| Bonten (31) 1996 | Ventilator-associated pneumonia: bronchoscopy with protected specimen brush (PSB) and bronchoalveolar lavage (BAL).  ICU mortality: mortality in ICU. |
| Steevens (32) 2002 | Diarrhea: stool volume ≥ 250 mL, or ≥ 3 loose stools per day.  Gastric residual volume: gastric aspirate ≥ 250 mL.  Aspiration: endotracheal tube aspirates with the presence of blue food coloring from enteral nutrition support (ENS) or visible gastric contents in pulmonary secretions. |
| Serpa (33) 2003 | Abdominal distension: abdominal circumference increased 3 cm or more.  Gastric residual volume: 150 mL or more of fluid could be aspirated immediately before each 3-hour feeding cycle.  Aspiration: inclusion of aniline blue dye marker in the diet, suctioned out within the endotracheal tube.  ICU mortality: mortality in ICU.  Length of ICU stay: number of days stay in ICU.  Diarrhea, Vomiting: lack of a clear definition. |
| Chen (34) 2006 | Gastric residual volume: gastric aspirate > 60 mL.  Aspiration pneumonia: the presence of a constellation of two or more symptoms within a three-day period defines a positive result (glucose in sputum, chest radiography, wheezing, fever). |
| MacLeod (35) 2007 | ICU mortality: mortality in ICU.  Length of ICU stay: number of days stay in ICU.  Achieved energy intake: percentage of the total required calories.  Pneumonia: Centers for Disease Control and Prevention (CDC) definition.  Diarrhea: lack of a clear definition. |
| Kadamani (18) 2014 | Aspiration: First, continuous assessment for the presence of oxygen saturation decline (< 90%), tachycardia (> 100 beats/minute), and central cyanosis. Second, testing for the presence of blue food dye in tracheal secretions.  Gastric residual volume: gastric aspirate > 200 mL.  Diarrhea: the patient passed 3 or more times of loose stool per day.  Constipation: the patient had absent bowel movement for three consecutive days or more.  ICU mortality: mortality in ICU.  Vomiting: lack of a clear definition. |
| Tavares de Araujo (36) 2014 | Diarrhea, Vomiting, Abdominal distension, Constipation: lack of a clear definition. |
| Mazaherpur (37) 2016 | Achieved energy intake: mean percentage of total energy. |
| McNelly (16) 2020 | Diarrhea: Bristol Stool Chart score ≥ 5 (loose or liquid).  ICU mortality: mortality in ICU.  Length of ICU stay: number of days stay in ICU.  Achieved energy intake: percentage of the energy.  Vomiting, Abdominal distension: lack of a clear definition. |
| Rana (38) 2021 | Gastric residual volume: gastric aspirate > 200 mL.  Diarrhea, Vomiting, Aspiration: lack of a clear definition. |
| Ren (39) 2021 | ICU mortality: mortality in ICU.  Length of ICU stay: number of days stay in ICU. |
| Lee (17) 2022 | Diarrhea: stool volume > 200 mL per day.  Constipation: more than 3 days without stool.  Vomiting: the detection of gastric contents in the oropharynx or on the external mouth.  Aspiration: the presence of food material in the airway.  ICU mortality: mortality in ICU.  Abdominal distension: lack of a clear definition. |
| Banaei (19) 2022 | Gastric residual volume: gastric aspirate > 200 mL.  Length of ICU stay: number of days stay in ICU.  Diarrhea, Constipation, Vomiting, Aspiration, Abdominal distension: lack of a clear definition. |
| Wilkinson (40) 2023 | ICU mortality: mortality in ICU. |
| Panwar (41) 2024 | ICU mortality: mortality in ICU.  Length of ICU stay: number of days stay in ICU. |
| Hrdy (42) 2025 | Diarrhea: passage of loose stools three or more times per day, with a volume exceeding 1 L/day.  Aspiration: the presence of enteral nutrition in the patient’s airway during standard airway care.  ICU mortality: mortality in ICU.  Length of ICU stay: the day of admission and discharge was counted as one.  Ventilator-associated pneumonia, Vomiting: lack of a clear definition. |
| Yao (43) 2025 | Gastric residual volume: gastric aspirate ≥ 250 mL.  ICU mortality: mortality in ICU.  Length of ICU stay: number of days stay in ICU. |

Abbreviations: ICU, intensive care unit.

**Supplementary Table 4. Subgroup Meta-Analysis**

| Outcome | Subgroup Dimension | Subgroup | No. of Studies | Effect Estimate (95% CI) | *I²* (%) | *P* |
| --- | --- | --- | --- | --- | --- | --- |
| Diarrhea | Overall | Overall Pooling | 10 | 0.77 (0.55-1.07) | 1.2 | 0.427 |
|  | Study Duration | < 7 days | 5 | 0.56 (0.32-0.98) | 0.0 | 0.595 |
|  |  | ≥ 7 days | 5 | 0.87 (0.52-1.46) | 9.3 | 0.353 |
| Constipation | Overall | Overall Pooling | 4 | 1.40 (1.01-1.95) | 4.2 | 0.372 |
|  | Study Duration | < 7 days | 2 | 2.55 (1.15-5.69) | 0.0 | 0.465 |
|  |  | ≥ 7 days | 2 | 1.25 (0.89-1.75) | 0.0 | 0.988 |
| Vomiting | Overall | Overall Pooling | 8 | 1.05 (0.59-1.87) | 36.3 | 0.139 |
|  | Study Duration | < 7 days | 5 | 0.94 (0.49-1.79) | 0.0 | 0.780 |
|  |  | ≥ 7 days | 3 | 1.07 (0.32-3.58) | 77.8 | 0.011 |
| Gastric residual volume | Overall | Overall Pooling | 7 | 0.80 (0.40-1.58) | 41.1 | 0.117 |
|  | Study Duration | < 7 days | 3 | 0.76 (0.31-1.89) | 0.0 | 0.607 |
|  |  | ≥ 7 days | 4 | 0.87 (0.30-2.56) | 67.2 | 0.027 |
| Abdominal distension | Overall | Overall Pooling | 5 | 0.77 (0.44-1.36) | 36.0 | 0.181 |
|  | Study Duration | < 7 days | 2 | 0.88 (0.56-1.39) | 0.0 | 0.319 |
|  |  | ≥ 7 days | 3 | 0.56 (0.16-1.96) | 62.0 | 0.072 |
| Aspiration | Overall | Overall Pooling | 7 | 0.88 (0.25-3.09) | 0.0 | 0.665 |
|  | Study Duration | < 7 days | 4 | 1.45 (0.23-8.96) | 0.0 | 0.957 |
|  |  | ≥ 7 days | 3 | 0.57 (0.06-5.10) | 37.9 | 0.200 |
| ICU mortality | Overall | Overall Pooling | 11 | 0.97 (0.78-1.20) | 0.0 | 0.475 |
|  | Study Duration | < 7 days | 3 | 1.12 (0.80-1.59) | 0.0 | 0.984 |
|  |  | ≥ 7 days | 8 | 0.90 (0.66-1.22) | 17.1 | 0.295 |
| Length of ICU stay | Overall | Overall Pooling | 8 | -0.04 (-0.16-0.09) | 0.0 | 0.943 |
|  | Study Duration | < 7 days | 2 | -0.04 (-0.25-0.18) | 0.0 | 0.899 |
|  |  | ≥ 7 days | 6 | -0.04 (-0.19-0.12) | 0.0 | 0.813 |

Abbreviations: CI, confidence interval; *I²*, heterogeneity index; *P*, *P*-value for subgroup interaction; ICU, intensive care unit.

The subgroup dimension is study duration (< 7 vs. ≥ 7 days); the “overall” estimate represents the pooled effect across all studies.

Effect sizes are presented as relative risk (RR) for dichotomous outcomes and standardized mean difference (SMD) for the continuous outcome Length of ICU stay in the Effect Estimate (95% CI) column.

**Supplementary Table 5. GRADE Assessment of Outcome Indicators**

| **Quality assessment** | | | | | | **No of patients** | | **Effect** | **Quality** | **Importance** |
| --- | --- | --- | --- | --- | --- | --- | --- | --- | --- | --- |
| No of studies | Risk of bias | Inconsistency | Indirectness | Imprecision | Other considerations | C | I | Relative  (95% CI) |  |  |
| Diarrhea | | | | | | | | | | |
| 10 | serious^1^ | no serious inconsistency | no serious indirectness | very serious^2^ | none | 48/460 | 63/447 | RR 0.77 (0.55 to 1.07) | VERY LOW | CRITICAL |
| Constipation | | | | | | | | | | |
| 4 | serious^1^ | no serious inconsistency | no serious indirectness | serious^3^ | none | 59/119 | 37/107 | RR 1.40 (1.01 to 1.95) | LOW | CRITICAL |
| Vomiting | | | | | | | | | | |
| 8 | serious^1^ | no serious inconsistency | no serious indirectness | very serious^2^ | none | 43/370 | 37/359 | RR 1.05 (0.59 to 1.87) | VERY LOW | CRITICAL |
| Gastric residual volume | | | | | | | | | | |
| 7 | serious^1^ | no serious inconsistency | no serious indirectness | very serious^2^ | none | 25/219 | 30/214 | RR 0.80 (0.40 to 1.58) | VERY LOW | CRITICAL |
| Aspiration | | | | | | | | | | |
| 7 | serious^1^ | no serious inconsistency | no serious indirectness | very serious^2^ | none | 3/297 | 4/288 | RR 0.88 (0.25 to 3.09) | VERY LOW | CRITICAL |
| Abdominal distension | | | | | | | | | | |
| 5 | serious^1^ | no serious inconsistency | no serious indirectness | very serious^2^ | none | 31/177 | 36/168 | RR 0.77 (0.44 to 1.36) | VERY LOW | CRITICAL |
| ICU mortality | | | | | | | | | | |
| 11 | serious^1^ | no serious inconsistency | no serious indirectness | very serious^2^ | none | 126/590 | 130/593 | RR 0.97 (0.78 to 1.20) | VERY LOW | IMPORTANT |
| Length of ICU stay | | | | | | | | | | |
| 8 | serious^1^ | no serious inconsistency | no serious indirectness | very serious^2^ | none | 491 | 484 | SMD -0.04 (-0.16 to 0.09) | VERY LOW | IMPORTANT |

Abbreviations: CI, confidence interval; RR, relative risk; ICU, intensive care unit; C, continuous enteral feeding; I, intermittent enteral feeding.

^1^ Blind method and allocation concealment are missing.
^2^ Small sample size and RR crossing the line of no effect.
^3^ The sample size is small.

**
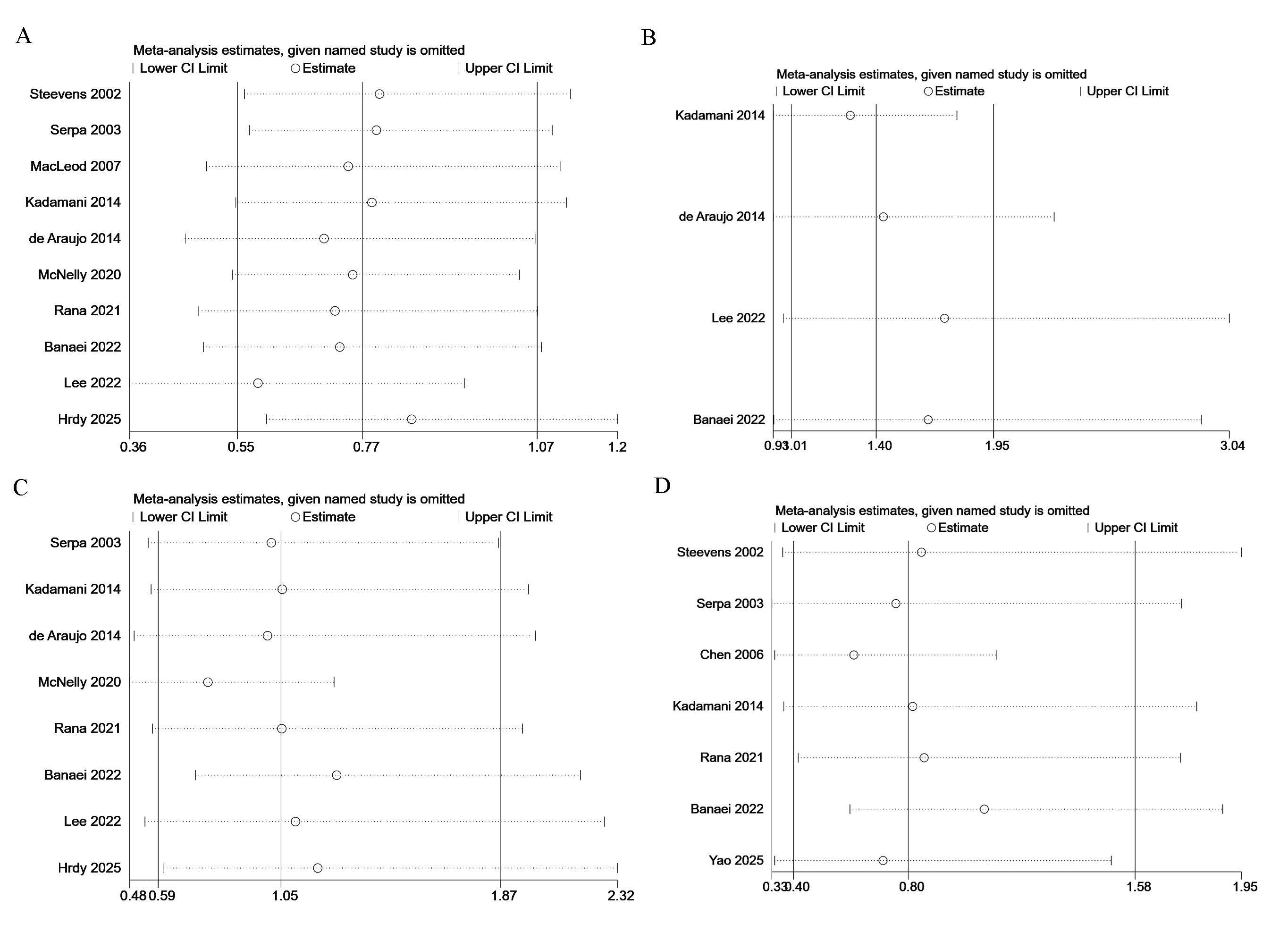
**

**Supplementary Figure 1.** Sensitivity analysis comparing continuous versus intermittent enteral feeding on feeding tolerance outcomes: **(A)** diarrhea, **(B)** constipation, **(C)** vomiting, and **(D)** gastric residual volume.

**
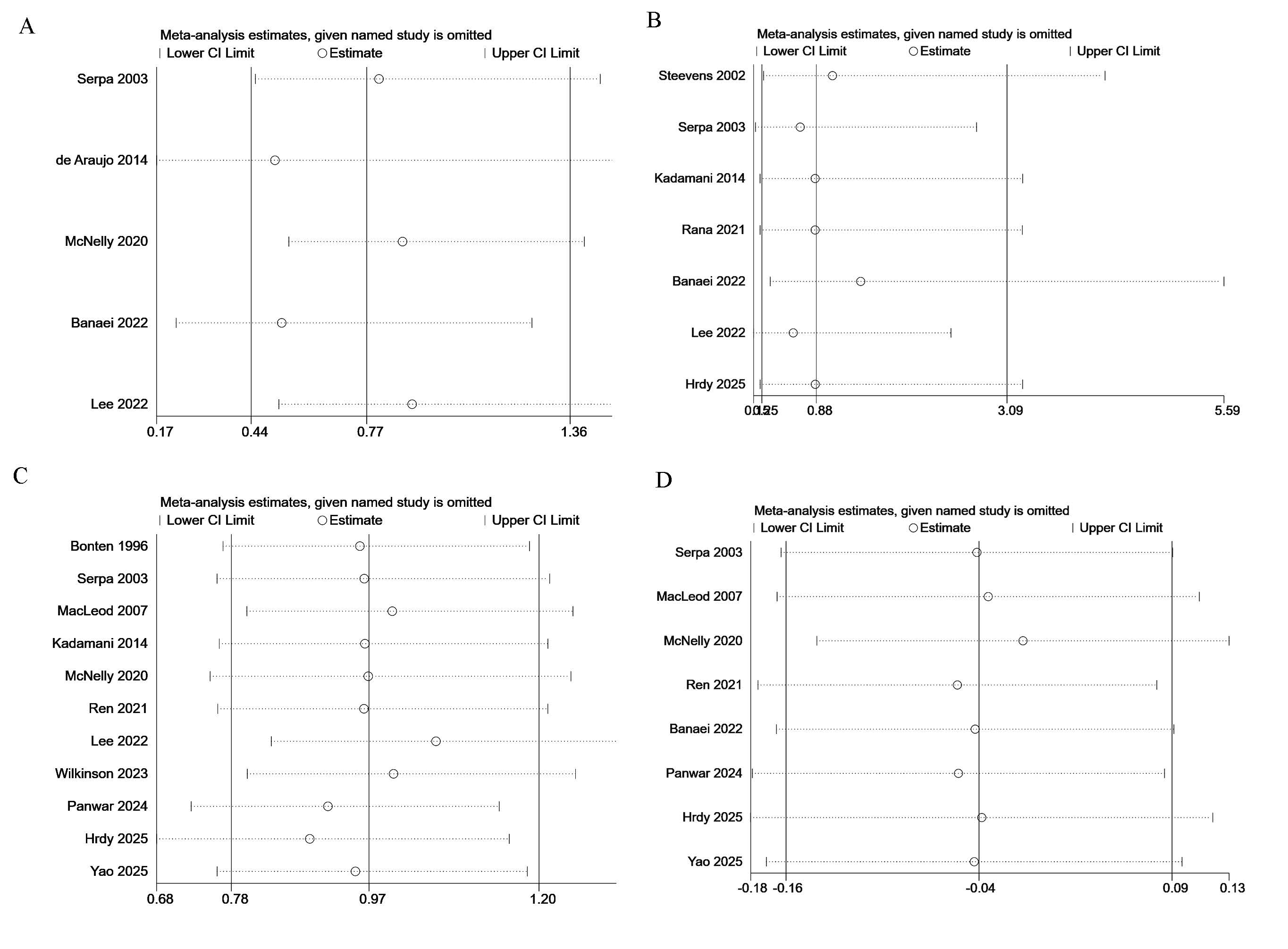
**

**Supplementary Figure 2.** Sensitivity analysis comparing continuous versus intermittent enteral feeding on clinical outcomes: **(A)** abdominal distension, **(B)** aspiration, **(C)** ICU mortality, and **(D)** length of ICU stay.

**
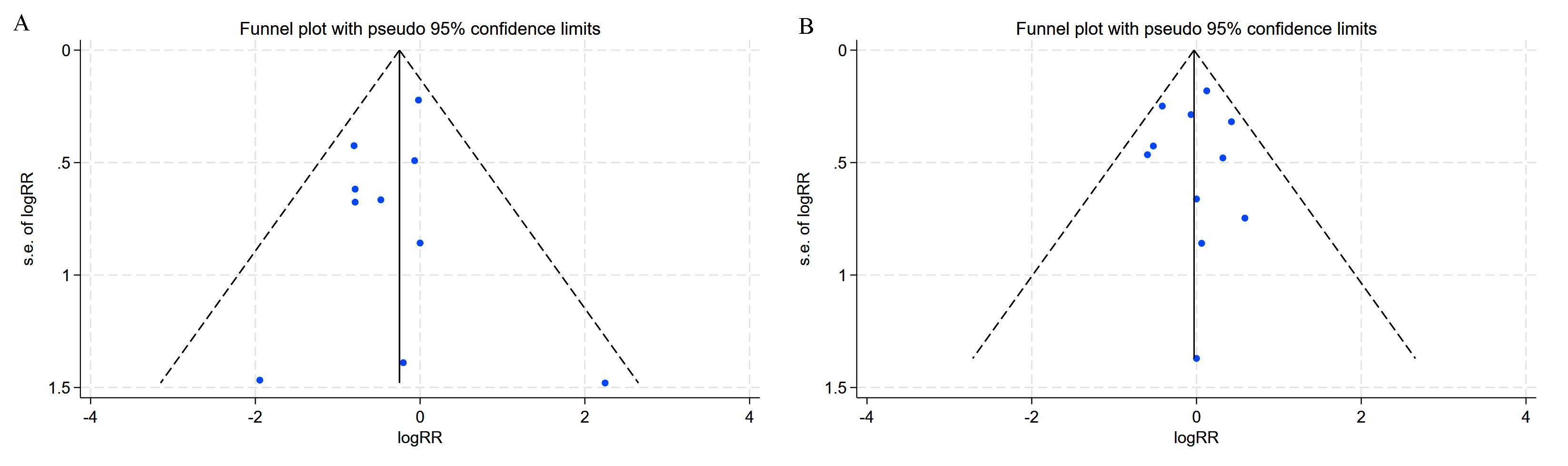
**

**Supplementary Figure 3.** Funnel plots for assessing publication bias: **(A)** diarrhea and **(B)** ICU mortality.
